# High-Pressure Microfluidics for Ultra-Fast Microbial Phenotyping

**DOI:** 10.3389/fmicb.2022.866681

**Published:** 2022-05-23

**Authors:** Anaïs Cario, Marina Larzillière, Olivier Nguyen, Karine Alain, Samuel Marre

**Affiliations:** ^1^Univ. Bordeaux, CNRS, Bordeaux INP, ICMCB, UMR 5026, Pessac, France; ^2^CNRS, Univ. Brest, Ifremer, IRP 1211 MicrobSea, Unité de Biologie et Ecologie des Ecosystèmes Marins Profonds BEEP, IUEM, Plouzané, France

**Keywords:** high-pressure microfluidics, deep-sea microorganisms, real time investigations, phenotyping, fast screening

## Abstract

Here, we present a novel methodology based on high-pressure microfluidics to rapidly perform temperature-based phenotyping of microbial strains from deep-sea environments. The main advantage concerns the multiple on-chip temperature conditions that can be achieved in a single experiment at pressures representative of the deep-sea, overcoming the conventional limitations of large-scale batch metal reactors to conduct fast screening investigations. We monitored the growth of the model strain *Thermococcus barophilus* over 40 temperature and pressure conditions, without any decompression, in only 1 week, whereas it takes weeks or months with conventional approaches. The results are later compared with data from the literature. An additional example is also shown for a hydrogenotrophic methanogen strain (*Methanothermococcus thermolithotrophicus*), demonstrating the robustness of the methodology. These microfluidic tools can be used in laboratories to accelerate characterizations of new isolated species, changing the widely accepted paradigm that high-pressure microbiology experiments are time-consuming.

## Introduction

The study of the deep biosphere is particularly concerned with the discovery and investigation of the deep microbial life inhabiting this remote environment. The case of marine environment is particularly interesting since if we consider that the deep biosphere begins in the ocean at depths greater than 1,000 m (Jannasch and Taylor, 1984) the deep-sea represents 65% of the Earth’s surface and 95% of its habitable space. The deep biosphere is estimated to account for more than ~15% of the total biomass on Earth (Whitman et al., 1998; Bar-On et al., 2018), and is known to be a major contributor to biogeochemical cycles (D'Hondt et al., 2004; Kallmeyer et al., 2012). In recent years, the deep ocean has been the focus of much attention because it hosts various ecosystems, contains biological resources—especially microbial—and mineral resources, and plays key functions for our planet (e.g., carbon storage, climate regulation, microbial degradation, receptacle of pollutants, etc.). However, despite its importance, the deep-sea remains sparsely documented, as does the deep microbial biosphere (Ramirez-Llodra et al., 2010). Knowledge of the deep biosphere is notably hampered by the difficulties inherent to its access, the technical difficulties to sample it, and the investigations at laboratory scale under realistic pressure conditions (up to 100 MPa), which require adapted equipment (Cario et al., 2019; Garel et al., 2019). Therefore, phenotyping (i.e., determining the detectable physical and biochemical characteristics of an organism or microbial strain) of microbial strains from deep-sea environments is time-consuming, given the large number of long and distinct experiments that must be performed—and repeated—in order to characterize a new strain, a mutant or simply to perform basic growth studies.

Since microorganisms in the deep biosphere experience diverse and severe conditions (Jørgensen and Boetius, 2007; Orcutt et al., 2013), characterization of microbial species at the laboratory scale requires specific high-pressure equipment and robust methodologies. Various culture-based and culture-independent approaches have attempted to describe both the microbial diversity and the array of metabolic capabilities present in these singular ecosystems, but with more effort devoted to omics-based studies in recent years at the expense of time-consuming culture-based approaches (Connon and Giovannoni, 2002; DeSantis et al., 2007; Rinke et al., 2013; Chen et al., 2021). The cutting-edge approaches for isolation and cultivation of prokaryotes such as microencapsulation, single-cell and droplet-based cultivation, and cell sorting based cultivation (e.g., reverse genomics; Zengler et al., 2005; Nichols et al., 2010; Boitard et al., 2015; Jiang et al., 2016; Berdy et al., 2017; Terekhov et al., 2018; Hu et al., 2020; Lewis et al., 2021) are very promising but cannot be applied to all microbial taxa as they do not mimic all environmental conditions, and in particular high temperature and pressure. Despite these significant achievements, the vast majority of the genera and phyla of bacteria and archaea on Earth, including those from the deep biosphere, do not have cultured representatives (Whitman et al., 1998; Lloyd et al., 2018). The low cultivation scores can be explained in part by the fact that many cultures are grown at atmospheric pressure, whereas in their *in situ* habitat, microorganisms are subjected to high hydrostatic pressures. Together with temperature and chemistry, pressure is one of the parameters driving the distribution and the microbial activities in the biosphere (Fry et al., 2008; Reith, 2011; Picard and Daniel, 2013; Jebbar et al., 2015) and is of great importance to isolate new strains and to study their physiology. Having a larger number of deep biosphere isolates grown under different catabolic conditions and characterized in terms of temperature range and growth pressure would shed light on their putative function in their natural habitat and in the geochemical cycles of their ecosystem.

To reproduce the extreme conditions of pressure, temperature and geochemistry at the laboratory scale, the conventional equipment is large reactors (from a few tens of milliliters to a few liters) made of stainless steel or other metals (e.g., Inconel, Hastelloy, titanium alloys, etc.), which have demonstrated excellent thermo-mechanical properties along with acceptable chemical compatibility and bio compatible properties (Kato et al., 1996; Deusner et al., 2010; Czernichowski-Lauriol et al., 2017; Garel et al., 2019). Such reactors are used to achieve desired culture conditions representative of the deep biosphere environment to mimic the conditions encountered by deep environment microbes, while beneficiating from a laboratory-scale environment (Yayanos, 1986; Jannasch et al., 1996; Nauhaus et al., 2002; Kato, 2011). Although several new and/or updated sampling equipment and bioreactors have been developed allowing pressure and temperature conditions to be maintained (Garel et al., 2019; Peoples et al., 2019), there is still a lack of research equipment that allows for both (i) pressure-retention during analysis, (ii) ability to implement *in situ* characterization techniques—such as simple visualization, and (iii) fast screening capabilities. Several recent developments have overcome some of these limitations by allowing equi-pressure transfer of samples from reactor to reactor (Garel et al., 2019; Oliver et al., 2021) or by inserting sapphire windows within metal reactors, allowing performing microscopic or spectroscopy investigations (Bao et al., 2010; Maldonado et al., 2016). Such developments have somehow reduced the need for depressurization during analysis but remain scattered. Besides, some limitations remain: large-scale reactors can generate undesirable gradients—temperature, chemical composition—which could induce a bias in the analysis. In addition, these experimental set-ups do not allow for rapid screening experimentation, generally requiring weeks or even months to fully characterize a strain, which significantly slows the speed at which phenotyping can be performed. Therefore, the field of high-pressure microbiology needs to focus on new approaches and instruments to overcome the classical limitations of culturing and studying deep biosphere microorganisms, which are critical for advancing environmental microbiological research. An interesting option to address this gap involves the use of microfluidic reactors.

Over the past 20 years, microfluidics has greatly contributed to the progress of (micro)biology research, including the integration of analytical techniques (Demello, 2006) with cell handling (El-Ali et al., 2006), biochemical assays, which have been dedicated to various research areas such as medical investigations (Sackmann et al., 2014), environmental microbiology (Dusny and Schmid, 2015; Kaminski et al., 2016), etc. These tools offer several advantages over conventional large-scale devices. Indeed, microreactors offer a solution for temperature and feed flow control, reproducibility, *in situ* monitoring (Demello, 2006), rapid parameter screening (Jensen et al., 2000), fast mass and heat transfer (Gervais and Jensen, 2006), and low sample consumption. The combination of size reduction, single-phase and/or multi-phase *in situ* flows and improved reliability, can be recorded in a fast screening methodology development policy. Indeed, microfluidics generates a large amount of data and is already used as a high-throughput cultivation platform for conventional microbiology (i.e., non-extremophilic microorganisms) where hundreds to thousands of microbial colonies at a single-cell level can be studied by automated time-lapse microscopy, leading to a better understanding of microbial behavior and physiology (Moffitt et al., 2012; Grünberger et al., 2015). Conventional microfluidics uses PDMS (polydimethylsiloxane) materials, a polymer that is not compatible with extreme cultivation conditions (e.g., anoxic, high salinity, and high-pressure conditions). Nevertheless, microreactors have been already used to study environmental soil microbiology (Pucetaite et al., 2021) but also marine microbiology, under normal conditions (i.e., non-extremophilic) using droplet-based microfluidics (Girault et al., 2019). Meanwhile, technological developments have been made to use microfluidics for geochemical and microbiological investigations in deep-sea waters, considering microreactors as *in situ* (bio)chemical sensors/detectors (Wang et al., 2009; Beaton et al., 2022). In that case, the microreactor undergoes isostatic pressure both externally and internally, therefore not requiring mechanically resistant materials for its fabrication. Besides, microflows have also permitted to develop flow cytometry (Adan et al., 2017) at single cell level for the sorting and further identification of strains in complex environmental samples using either shape and/or fluorescence (fluorescent labeling molecules) detection, although no demonstration has been reported so far for high-pressure conditions. In order to extend the range of microfluidics to high-pressure microbiology investigations as well as deep environmental microbiology at laboratory scale, two strategies have been considered. First, researchers have used transparent capillary tubings (e.g., glass, silica, and sapphire), either with circular or square crossed section, for studying deep environment microorganisms using visible or fluorescence imaging (Beney et al., 1997; Raber et al., 2006; Bourges et al., 2020). Such capillaries offer affordable and easy strategies to access microbial characterization under high pressure, up to 100 MPa. Secondly, the use of diamond anvil cell or specific high-pressure cells, combined to spectroscopy techniques are also used for *in situ* monitoring and characterization studies under high-pressure conditions (Kato and Fujisawa, 1998; Molina-Gutierrez et al., 2002; Oger et al., 2006; Picard et al., 2007; Peters et al., 2014) but are not yet widespread because of specific and costly equipment.

Despite their ease of implementation, capillaries suffer from difficulties in accessing elaborate fluidic designs and controlling temperature. Therefore, it is difficult to envision their use for high throughput screening studies, in particular, for microbial phenotyping.

To address these limitations, on chip high-pressure microreactors have been recently developed (Marre et al., 2010) and utilized for instance for thermodynamics investigations (Xu et al., 2017; Gavoille et al., 2019) or deep underground fluids flow and geochemistry studies (Morais et al., 2016, 2020). High-pressure microreactors can be made out of silicon and Pyrex (semi-transparent), glass–glass (Murphy et al., 2007; Tiggelaar et al., 2007), or even sapphire (i.e., fully transparent; Marre et al., 2021). Such tools benefit from all the advantages of microfluidics, while being compatible with the representative pressure and temperature conditions inherent to deep environment. With the same ability than their room pressure counter-parts, high-pressure on-chip microreactors provide the ability to screen multiple conditions in a single experiment, paving the way for the study of the deep biosphere in the laboratory using new rapid screening methodologies.

In this article, we demonstrate for the first time the use of a novel transparent, high-pressure, biocompatible microfluidic platform for ultra-fast screening of temperature-dependent growth conditions of microorganisms from deep environments. The concept is demonstrated using two marine species with different metabolic strategies: *Thermococcus barophilus* MP^T^ (Marteinsson et al., 1999), a model piezo-hyperthermophilic and heterotrophic strain isolated from a deep-sea hydrothermal vent and *Methanothermococcus thermolithotrophicus*, a thermophilic and a model hydrogenotrophic methanogen strain isolated from a seafloor geothermal spring (Huber et al., 1982). After introducing the overall methodology, design and fabrication of the microreactor, we present the investigation protocol and the obtained results, which are compared to the literature data.

## Materials and Methods

### Model Strains and Growth Media

We detailed hereafter the two considered strains along with the associated growth media, which were used in this study.

#### Thermococcus barophilus

*Thermococcus barophilus* strain MP^T^ (DSM 11836) was obtained from the DSMZ collection (Deutsche Sammlung von Mikroorganismen und Zellkulturen, Braunschweig, Germany). It is a piezo-hyperthermophilic strain, which was isolated from a deep-sea hydrothermal vent (Snake Pit) on the Mid-Atlantic Ridge (depth, 3,550 m; Marteinsson et al., 1999). It grows from ambient pressure to 80 MPa and exhibits an optimal temperature for growth of 85°C over a wide pressure range (0.3–40 MPa; Marteinsson et al., 1999; Zeng et al., 2009). In this study, the *T. barophilus* cultures were carried out in a *Thermococcales* Rich Medium (TRM, pH 6.8; Zeng et al., 2009), under anoxic conditions. After autoclaving at 121°C during 30 min, the medium was aliquoted into several sterile Hungate tubes (10 ml), sealed with butyl rubber septa, reduced by adding 0.5% reductant (v/v, polysulfides stock solution of 0.05 M; Ikeda et al., 1972), and placed under an inert atmosphere (Ar, 30 kPa). The reductant was also used as a soluble source of sulfur for *T. barophilus* cells.

#### Methanothermococcus thermolithotrophicus

*Methanothermococcus thermolithotrophicus* strain SN-1^T^ (DSM 2095) was obtained from the DSMZ collection (Deutsche Sammlung von Mikroorganismen und Zellkulturen, Braunschweig, Germany). It is a thermophilic and hydrogenotrophic methanogen archaeon isolated from a geothermally heated seafloor in Italy (Huber et al., 1982). This strain is able to grow on a wide temperature range, from 30°C to 70°C (Huber et al., 1982) with an optimum temperature at 65°C, and displays a piezophilic behavior when growing under elevated pressure conditions (i.e., 50 MPa; Bernhardt et al., 1988). In this study, *M. thermolithotrophicus* was cultivated in the AGW (Artificial Ground Water) medium (Dupraz et al., 2013) supplemented with 120 mM HEPES. The anoxic cultures were performed optimally at 65°C in serum flasks and with a 0.2 MPa gas mixture (mol/mol) of 80% H_2_ and 20% CO_2_, with a final cell density in stationary phase reaching 2×10^8^ cells.ml^−1^. When growing at elevated pressure conditions, the H_2_/CO_2_ partial pressure was established either at 2 or 3 MPa and supplemented with inert gas (Nitrogen) to reach the desired total pressure in the set up (i.e., 5 and 10 MPa, respectively). Only the initial (T_0_) and end-points (T = 18 h of growth) were considered to evaluate the growth of this strain into the high-pressure microfluidic temperature gradient setup.

In all cases, the pre-cultures in mid-exponential phase of growth were used to inoculate Hungate tube and then transferred to the high-pressure microfluidic reactors with an initial cell concentration of ~5.10^6^ cells.ml^−1^. In the case of *T. barophilus*, for which a full phenotyping study was performed, cell concentrations in control culture tubes at 85°C (Hungate tube for ambient pressure condition and high-pressure vessels for high-pressure conditions) were assessed by direct cell counting onto a Thoma chamber (Preciss, France; surface area: 0.0025 mm^2^, depth: 0.1 mm) using a DM2000 LED phase contrast optical microscope (Leica Microsystems CMS GmbH, Germany).

### Microreactor Design and Set-up

The overall strategy for performing multi-temperature culture of microorganisms under pressure on a chip is to take advantage of microfluidic pools, which will serve as micro-wells, each exposed to a particular temperature condition. Microbial growth can then be monitored directly *in situ* for each pool by microscopic counting. We detail hereafter the microreactor design and dimensioning strategy employed to achieve this goal.

**Figure 1:**
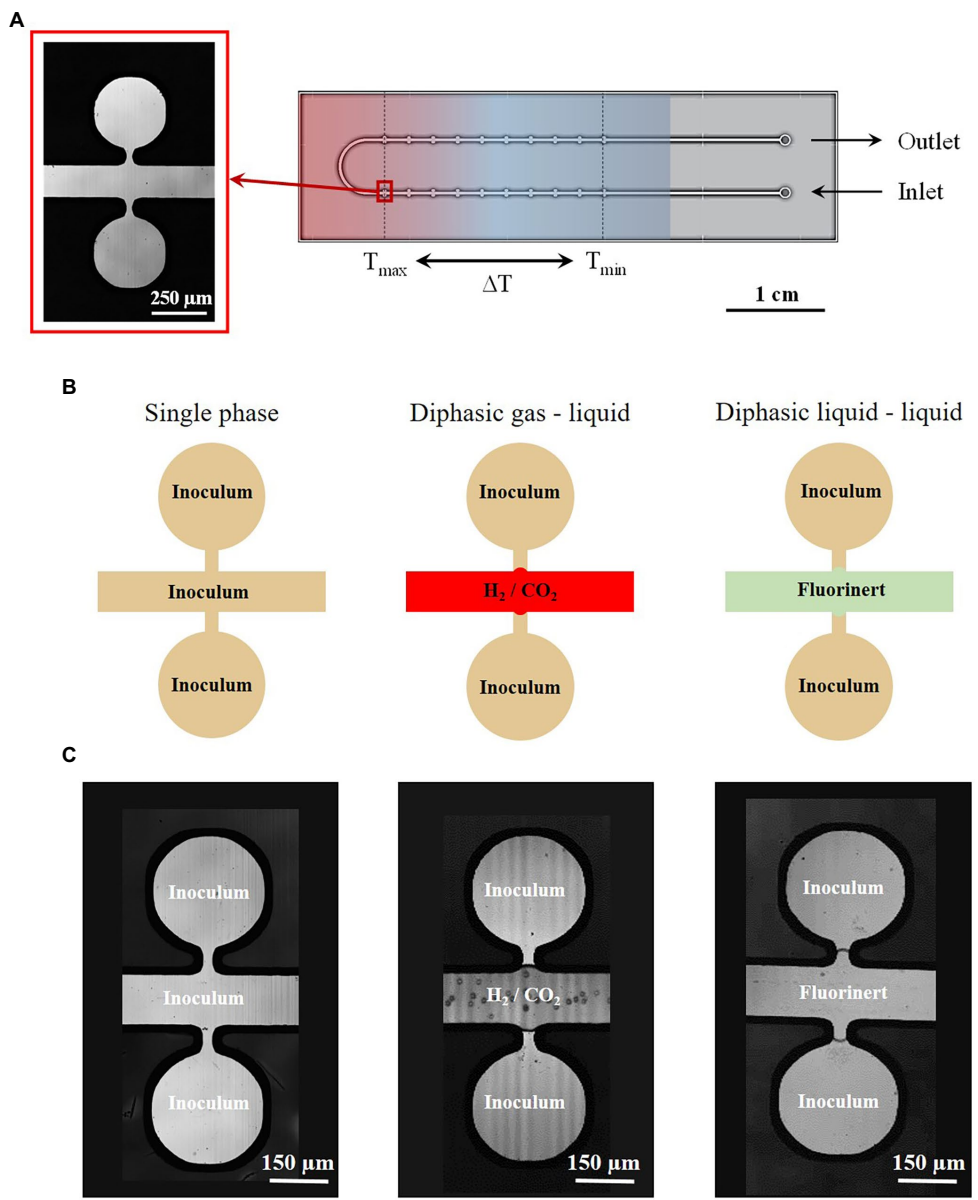
**(A)** Design of the temperature-gradient microreactor developed and used in this study with a microscope picture of the micropools used for both *Thermococcus barophilus* and *Methanothermococcus thermolithotrophicus* cultivation. **(B)** Different feeding strategies implemented into the microfluidic setup using growth medium phases and several interfaces. **(C)** Pictures of the concerning cultivation interfaces into the microreactor.

The microreactors were fabricated using the well-known silicon/Pyrex technology (Marre et al., 2010). This microfabrication technology is chosen for: (i) the optical access through the Pyrex side, allowing *in situ* characterization by optical microscopy, (ii) the excellent thermomechanical properties of silicon and Pyrex, allowing to work in a wide range of pressure and temperature conditions, and (iii) the biochemical inertness of both materials ensuring a good biocompatibility. The microreactors were fabricated using standard photolithography, wet etching and anodic bonding process, as previously described in the literature (Marre et al., 2010). The microreactor (Figure 1A) consists of one inlet and one outlet, used to inject the inoculated growth medium. It displays a U-shaped central microchannel (width: *w* = 200 μm, depth: *d* = 30 μm and length: *L* = 95 mm), which is used to deliver the fluid to 10 pairs of regularly spaced quasi-circular culture micropools on each branch, in which the microorganisms grow (diameter: *D* = 300 μm, depth: *d* = 30 μm, *V* = 1.5 nl or 1.9 nl, depending on the experiments). Each pool are placed on either side of the main channel and connected by a restriction channel (50 μm wide).

The main interest of this design stands in the possibility not only to work in a single phase mode were the inoculum occupied both the micro-chambers and the main channel but also to work in diphasic mode aiming at using a fluid non miscible with the inoculum for both “sealing” and feeding the micro-chambers with molecules through phase transfer (Figure 1B). In these cases, both liquids and gases may be considered. The microreactor surface being hydrophilic, the wetting phase is the inoculum. Hence, the wettability properties favor the inoculum to stay inside the micro-chamber (Figure 1B). However, the restriction channels are used to prevent any invasion of the micro-chambers with the second phase due to fluid flows during the microreactor filling procedure.

**Figure 2:**
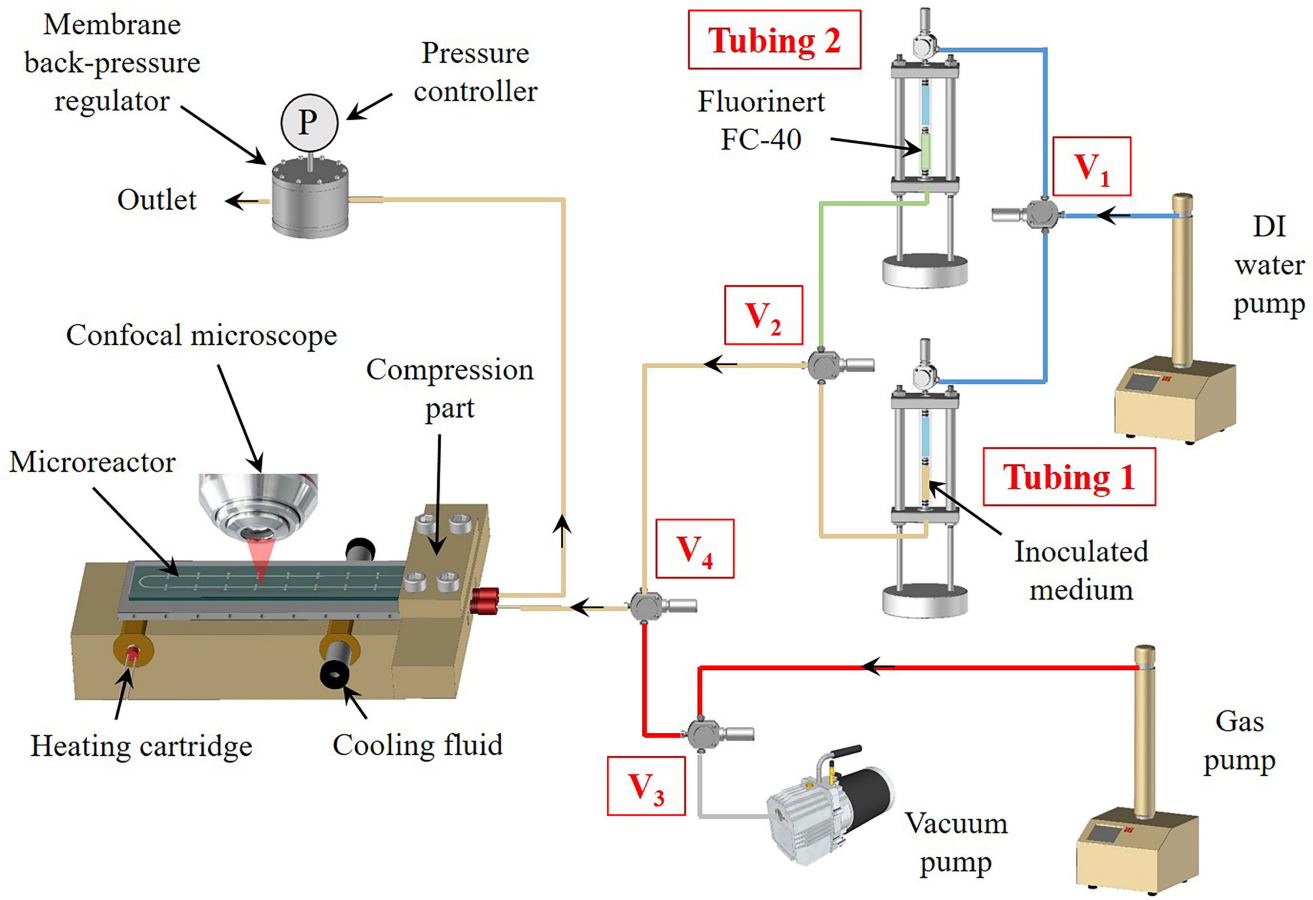
Set-up of the high-pressure microfluidics platform for phenotyping microorganisms at temperature (see the description in the text).

The microreactor is in contact with a temperature gradient generating device ensuring a linear temperature gradient between a T_min_ (either 60.1°C or 72.5°C for *M. thermolithotrophicus* and *T. barophilus*, respectively) and a T_max_ (either 70°C or 95°C for *M. thermolithotrophicus* and *T. barophilus*, respectively) along the microchip (Figure 2; Supplementary Information 1). Thus, 10 different temperatures could be examined simultaneously in a single experiment, while four different microchambers were subjected to the same temperature, providing a fourfold measurement to estimate the variability of results (Supplementary Figure S1). For *T. barophilus*, experiments were performed at three different pressures, 0.1, 5, and 10 MPa, in microreactors with 1.5 nl chambers. In parallel, experiments at 0.1 MPa (control) and 15 MPa were performed in microreactors with 1.9 nl chambers to demonstrate the robustness of the approach, independent of the microreactor itself. The microreactor with the 1.9 nl chambers was used for *M. thermolithotrophicus* experiments.

In a typical experimental set-up (Figure 2), the high-pressure temperature-gradient microreactor is connected to external fluid management equipment using a compression part, as described previously (Marre et al., 2010), made of polyetheretherketone (PEEK) for its biocompatibility characteristics and thermomechanical stability. The microreactor is placed on the temperature gradient system, which consists of an insulating block made of PEEK (for its thermal insulation properties) inside which two copper cylinders are inserted. A heating cartridge is inserted in the first cylinder, to generate a stable hot temperature zone using a Eurotherm temperature controller. The second cylinder is cooled to a targeted low temperature using a water flow provided by a circulating cryostat bath. To ensure a perfectly linear thermal gradient, an aluminum plate is placed in contact between the two copper cylinders, right below the microreactor (Figure 2; Supplementary Figure S1). A thermal paste is deposited between the aluminum and the microreactor to ensure excellent heat transfer. The whole assembly (i.e., microreactor + compression part + temperature gradient device) is placed under a confocal LASER scanning microscope. The compression part is connected to transparent tubes containing the fluids and equipped with movable pistons, in order to push the fluids into the microreactor.

### Experimental Protocol

We have used two distinct protocols depending on both the considered strain and their growth strategy. Each protocol corresponds to a specific method depending on whether we considered a single fluid, a diphasic liquid–liquid or a gas–liquid approach. In a typical high-pressure microfluidic experiment, the inoculation was performed in sterile and anoxic ways with either *T. barophilus* or *M. thermolithotrophicus*.

First, a Hungate tube containing 10 ml of either TRM medium supplemented with polysulfides for *T. barophilus* or AGW medium for *M. thermolithotrophicus* (see the growth medium above) was inoculated from a mid-exponential phase pre-culture to reach a final cell concentration of 5 × 10^6^ cells.ml^−1^. This cell concentration was chosen to ensure filling all the micropools in the microfluidic setup with 5–10 cells. Indeed, preliminary tests have shown that by considering a lower cell concentration, some micropools were empty. After inoculation, the Hungate tube was homogenized and 2.0 ml were transferred in a 5 ml high-pressure transparent tube equipped with a movable piston (Tubing 1, Figure 2), which had been first degassed (five cycles of N_2−_30 kPa/vacuum) to maintain anoxic conditions. This latter was then closed and connected *via* PEEK caps and connectors to a Teledyne ISCO high-pressure piston pump, previously filled with DI water as a pressurization fluid through a 3-way valve (V_1_, Figure 2). The pressure was applied by pushing with DI water on the mobile piston. The microreactor was first vacuumed to remove the air phase inside the device and later flushed with inert gas (N_2_) by opening valves V_3_ and V_4_ (Figure 2). This cycle was reproduced three times and the microreactor was finally left under vacuum to ensure getting rid of any gas bubbles in the micro-chambers during the filling procedure. Then, the microreactor was filled by delivering the inoculated growth medium at a flowrate of 50 μl.min^−1^ by opening V_2_ and V_4_ (Figure 2). The pressure inside the full set-up was maintained thanks to a membrane back pressure regulator (Equilibar) placed downstream the microreactor, enabling precise control of the pressure down to ultra-low flowrates (Figure 2).

When considering single-phase growth strategy (i.e., with *T. barophilus*), the pressure was slowly increased using the ISCO syringe pump (30 MPa.h^−1^) to the desired conditions. The pump was then set into a pressure constant mode at the working pressure. The remaining 8.0 ml of the inoculated medium was divided to serve as both a high-pressure positive control (4.0 ml corresponding to the high-pressure experimental pressure conditions) and a room pressure control (4.0 ml), at 85°C. In parallel, a 10-ml negative control (growth medium without inoculum) was performed to ensure there would be no contamination issues during the experiment.

When considering a diphasic gas–liquid growth strategy (i.e., with *M. thermolithotrophicus*), once the microreactor has been filled with the liquid inoculum, the pressure was set to the desired operating pressure and valve V_2_ was closed. Then, the gas phase (H_2_/CO_2_ mixture, 80/20 mol/mol, supplemented with N_2_ to reach the desired total pressure) was first pressurized inside the high-pressure syringe pump (Gas pump, Figure 2) up to the working pressure. Valve V_3_ was opened in order to inject the gas phase inside the microreactor at a slow flowrate (1 μl.min^−1^) to avoid any undesirable invasion of the microchambers by the gas phase, resulting in gas–liquid interfaces between the microchambers and the main microchannel (Figures 1B,C).

Potentially, in the case of a diphasic liquid/liquid growth strategy (not utilized in this study), the same procedure can be applied: first, a pressurization of the microreactor with the inoculum, followed by the injection of an immiscible liquid (e.g. Fluorinert FC40), through the switch of the three-way valves V_1_ and V_2_ to inject the Fluorinert from Tubing 2 (Figure 2) to the microreactor. Similarly, as for gases, a low flowrate is applied (1 μl.min^−1^) to avoid any invasion of the microchambers by the inert fluid (Figures 1B,C).

### Image Analysis: Pool Area Measurement and Cell Counting

**Figure 3:**
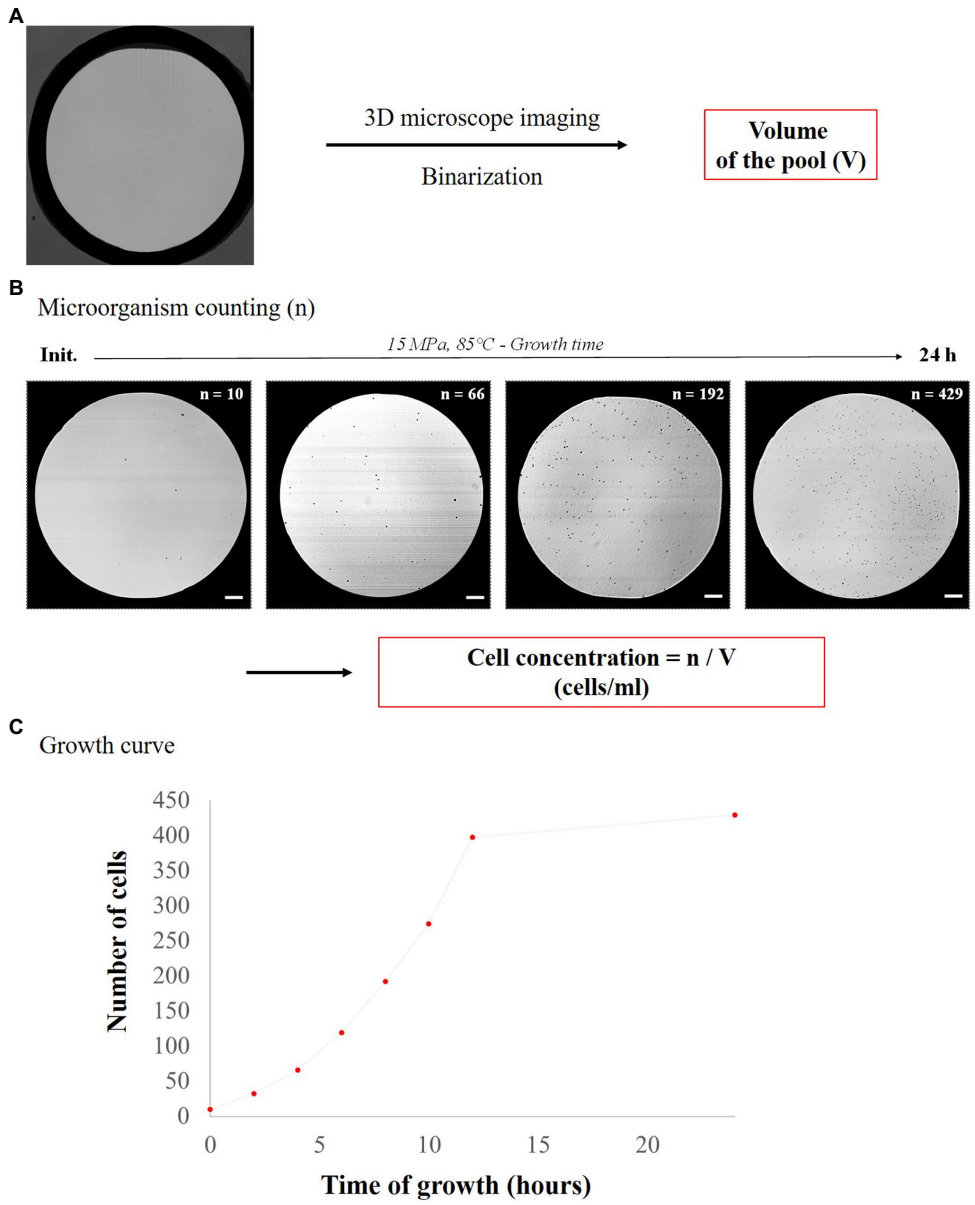
Schematic flowsheet for determination of cell concentration in each pool. **(A)** Determination of micropool volumes using 3D confocal microscopy and ImageJ analysis and **(B)** examples of pictures of *Thermococcus barophilus* growth over time with the corresponding cell number (*n*) at 15 MPa and 85°C (scale bar = 25 μm), allowing determination of cell concentration (cells.ml^−1^). **(C)** Growth curve with cell number versus time (15 MPa, 85°C).

**Figure 4:**
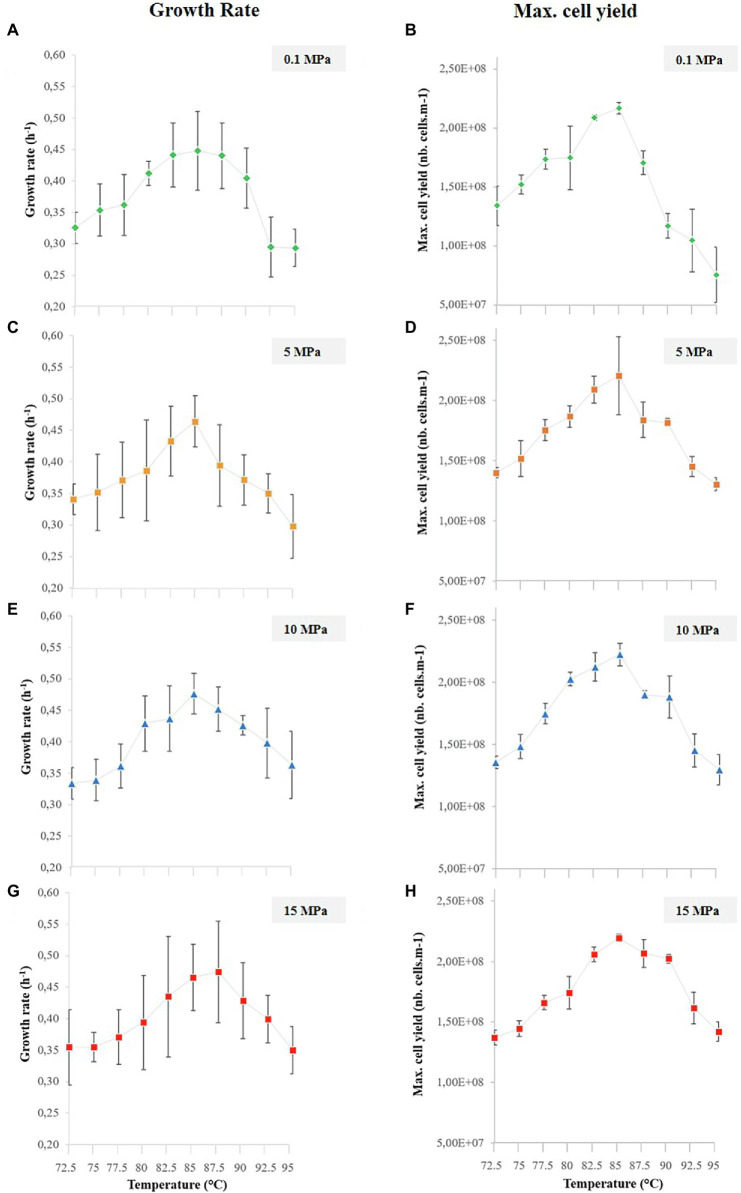
*Thermococcus barophilus* growth rates (left column) and maximal cell yields (right column) over 10 temperature conditions (i.e., 72.5°C–95°C) for several pressure conditions, while growing in the temperature gradient on-a-chip: **(A)** *T. barophilus* growth rates at 0.1 MPa. **(B)** *T. barophilus* maximum cell densities at 0.1 MPa. **(C)** *T. barophilus* growth rates at 5 MPa. **(D)** *T. barophilus* maximum cell densities at 5 MPa. **(E)** *T. barophilus* growth rates at 10 MPa. **(F)** *T. barophilus* maximum cell densities at 10 MPa. **(G)** *T. barophilus* growth rates at 15 MPa. **(H)** *T. barophilus* maximum cell densities at 15 MPa. Error bars represent the SD from the four replicates on a single experiment using the gradient on chip microfluidic setup.

**Figure 5:**
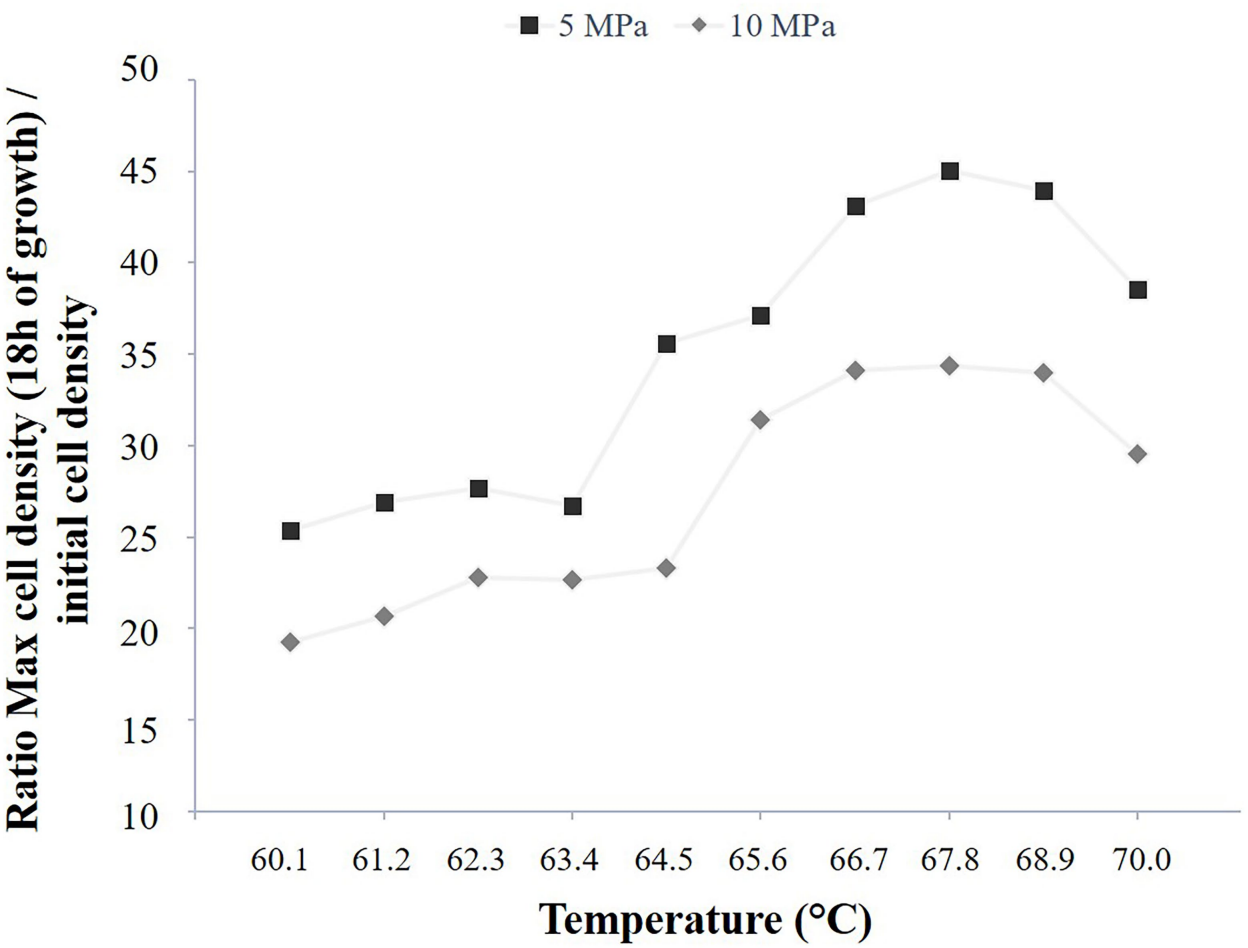
*Methanothermococcus thermolithotrophicus* growth comparison in a temperature gradient-on-a-chip between two-pressure conditions, 5 and 10 MPa total pressure (2 and 3 MPa of partial pressure of H_2_/CO_2_, respectively). Ratio of the mean cell density after 18 h of growth to the corresponding mean initial cell density, for two temperature gradient microfluidic experiments (temperature: 60.1°C–70°C).

The initial cell concentration (about 4–5 × 10^6^ cells.ml^−1^ depending on the micropool volume) was experimentally confirmed by counting the cells in each micropool. As a starting point, the number of cells per pool was typically 8 ± 3 cells. Then, the growth rate in each micropool was determined as follows (Figure 3A). First, the volume of each pool was accurately determined using 3D confocal imaging followed by image analysis (ImageJ©), as detailed in Supplementary Information 3. Cell counting overtime (Figure 3B) was then performed based on image captures made with a confocal microscope (Leica Microsystems, SP8), at 40× objective (in reflection mode) using the Leica LasX© software. OpenCFU software (Geissmann, 2013) was used for semi-automatic counting and for more accurate cell counting. Since both *T. barophilus* and *M. thermolithotrophicus* cells are tiny cocci (0.8–2.0 μm diameter; Huber et al., 1982; Marteinsson et al., 1999), the processing parameters of OpenCFU were adjusted to detect both species in the micro-chambers (see Supplementary Information 3 for details concerning the choosen OpenCFU parameters). Data were then combined to plot the growth curve for each micropool (Figure 3C), which all exhibit the classical shape of microbial growth curve (Supplementary Figure S2; i.e., lag phase, exponential phase and stationary phase—the death phase was not detected in here due to the “short” incubation time of 24 h max considered in our experiments). Finally, an extrapolation of the number of cells in the micropools was performed to obtain a normalized cell concentration (cells.ml^−1^) over time (Supplementary Figure S2). This extrapolation was done using the following formula:

*C* = *n*/*V.*

with *C* corresponding to the cell concentration (cells.ml^−1^), *V* to the volume of the micropool and *n* to the number of cells per pool. After conversion of cell concentrations to logarithmic values, growth rates were calculated from the logarithmic growth phase slopes of quadruplicate micropool culture experiments using the LINEST function in Excel. Error bars indicate the standard error from linear regressions of quadruplicate experiments (four micropools for one temperature data point) using the microfluidic temperature gradient chip (Figure 4). Maximum cell densities were measured from the stationary phase for all pressure conditions and error bars indicate standard deviation of quadruplicate experiments (Figure 4 for *T. barophilus* and Figure 5 for *M. thermolithotrophicus*). For *T. barophilus*, an overnight positive control (batch culture in 10 ml flask for atmospheric pressure and 5 ml for high-pressure millifluidic vessel under high-pressure conditions) at 85°C with the same cell inoculum as in the microfluidic experiment was always performed to ensure cell viability. Since the conventional high-pressure experiment requires decompression, only an endpoint measurement (after 12 h of growth) was considered for cell counting and compared to microfluidic growth at the same time-period, to avoid any growth bias due to decompression.

## Results

### *Thermococcus barophilus* Phenotyping

Images taken overtime showed an increasing number of cells in the micropools (up to 24 h of growth), regardless of the pressure or temperature considered. This allowed us to estimate the evolutionary growth of *T. barophilus* (Figures 3B,C, 4) as a function of temperature for different pressures. Each experiment is performed at a single pressure (0.1, 5, 10, and 15 MPa, respectively), but gathers 10 different temperature conditions (72.5°C–95°C) in quadruplicate (i.e., 40 micro-batch experiments in a single run). Based on these images, cell numbers were correlated to the corresponding cell concentrations in cells.ml^−1^, allowing the phenotype of *T. barophilus* to be screened under 40 different P/T conditions and obtaining 40 growth curves in only 1 week of experiments (Supplementary Figure S2).

To compare the combined effects of high pressure (without decompression) and temperature on *T. barophilus* growth already reviewed in the literature, we plotted the growth rates and maximum cell yields (Figure 4) obtained in each pressure experiments on the temperature gradient on-a-chip. Average growth rates and maximal cell yields correspond to replicate of four microbial growths in a single experiment. The average growth rates ranged from 0.29 ± 0.03 to 0.45 ± 0.06, 0.30 ± 0.05 to 0.46 ± 0.04, 0.33 ± 0.02 to 0.48 ± 0.03 h, and 0.35 ± 0.04 to 0.47 ± 0.05 h^−1^ for 0.1, 5, 10, and 15 MPa pressure conditions, respectively (Figures 4A,C,E,G left column). The temperature-dependent pattern was similar for all four pressure conditions: growth rates were the highest for a temperature close to the known optimal temperature for *T. barophilus* (i.e., 85°C; Marteinsson et al., 1999), and decreased when moving away from the optimum, which is in accordance with previously published data. It is interesting to note that, due to the large amount of data available, the growth rate curves could be plotted with slightly better accuracy (2.5°C shift between each condition) than that obtained with the classical approach. It can also be observed that the temperature evolution of the growth rates shifted slightly to higher temperature values when growth was performed at higher pressure conditions (5, 10, and 15 MPa), however, the optimal growth temperature was not affected by the pressure variations in the explored range.

This shift was also observed with the maximum cell yields which ranged from 7.54 ± 2.33 × 10^7^ to 2.17 ± 0.05 × 10^8^, 1.30 ± 0.05 × 10^8^ to 2.17 ± 0.32 × 10^8^, 1.30 ± 0.12 × 10^8^ to 2.22 ± 0.09 × 10^8^, and 1.37 ± 0.06 × 10^8^ to 2.19 ± 0.30 × 10^8^ cells.ml^−1^ for 0.1, 5, 10, and 15 MPa pressure conditions, respectively (Figures 4B,D,F,H right column). For comparison, parallel experiments in millifluidic high-pressure vessels were performed for each pressure at 85°C, as positive controls. *T. barophilus* growth was checked after one night of growth and reached the plateau of 2.10^8^ cells.ml^−1^ for each high-pressure experiment (data not shown), thus in good agreement with the results of the microfluidic approach.

### Experiments With *Methanothermococcus thermolithotrophicus*

The high-pressure microfluidic temperature gradient setup was also implemented with the cultivation of a methanogen strain in order to show the cultivation versatility of this setup. As the model strain is a thermophilic and hydrogenotrophic methanogen, it requires the supply of a gas phase (H_2_/CO_2_) to grow. The temperature range was chosen as a function of the literature (i.e., 60°C–70°C, Bernhardt et al., 1988) for two pressure conditions, 5 and 10 MPa (i.e., total pressure) with a partial gas pressure (H_2_/CO_2_, 80/20% mol/mol) of 2 MPa and 3 MPa, respectively. As this strain was previously described to better grow under elevated pressure conditions (Bernhardt et al., 1988), these partial pressures were chosen in order to screen rapidly the effects of dissolved gases (H_2_ and CO_2_) at 10 different temperatures on *M. thermolithotrophicus* growth (i.e., cell yield in stationary phase). To do so, a quadruplicate counting was performed for these 10 temperature conditions, both at the initial time point and after 18 h of growth, for both pressure conditions considered in this study (5 and 10 MPa; Figure 5; Supplementary Figure S3A).

Interestingly, the cell yields at 5 MPa (P_i_ = 2 MPa) reach the optimal cell density observed at atmospheric pressure conditions (about 2 × 10^8^ cells.ml^−1^) for both the optimum growth temperature and the above ones (i.e., 65°C–70°C; Supplementary Figure S3A). However, for growth temperatures below the optimum (i.e., 60.1°C–63.4°C), the cell yields reach a lower cell density, approximatively two-fold less (about 1.25×10^8^ cells.ml^−1^) with an intermediate temperature at 64.5°C (about 1.5 × 10^8^ cells.ml^−1^; Supplementary Figure S3A). When considering the initial cell density into the micropools, *M. thermolithotrophicus* cells seem to grow better at temperatures between 66.7°C and 68.9°C (Figure 5). The same growth temperature pattern is observed when *M. thermolithotrophicus* is cultivated at 10 MPa (Pi = 3 MPa). Indeed, the strain displays a higher cell yield when growing at temperatures between 65.6°C and 68.9°C. However, the highest cell density is 2-fold lower than the optimum cell density known for this strain (Figure 5; Supplementary Figure S3A).

## Discussion

In this study, we provide proof-of-concept for the operation of a novel high-pressure microfluidic culture approach and demonstrate that it can rapidly screen the effects of temperature on the growth of the piezo-hyperthermophilic model of deep hydrothermal origin *T. barophilus* strain MP^T^ (Marteinsson et al., 1999) along with a marine thermophile strain *Methanothermoccus thermolithotrophicus*. *Thermococcus barophilus* uses molecular and structural adaptation to thrive in its harsh environment, being able to cope with high pressure and fluctuating salinity and temperature conditions (Vannier et al., 2015; Cario et al., 2015a,b, 2016). This piezophilic model is easy to manipulate (Thiel et al., 2014) and is a versatile microorganism with unique metabolic attributes for adaptation to deep-sea vent conditions (Le Guellec et al., 2021). In addition, this archaeal species exhibits several other deep-sea vent clones with other metabolic properties (Kozhevnikova et al., 2016; Oger et al., 2016) expanding the catabolic capabilities of this species. Therefore, this strain was a prime model for this study. Meanwhile, *M. thermolithotrophicus* is a model methanogenic strain, already cultivated and studied under elevated pressure conditions (Bernhardt et al., 1988; Jaenicke et al., 1988; Miller et al., 1988).

Microorganisms in the deep biosphere flourish under extreme environmental conditions and only a small fraction of them have been isolated to date. Working on isolates remains a technical challenge but the best opportunity to understand the microbial mechanisms enabling life at depth and the limits of life on Earth. Studying growth behaviors under such extreme conditions provides insight into the molecular and cellular processes developed by piezophilic strains for microbial adaptation to high pressure. Recreating the *in situ* pressure conditions require specific culture approaches and is extremely time-consuming for strain phenotyping. Classical microbiology approaches have overcome some of these time issues by using spectrophotometry and other automated counting methods (e.g. cell sorting and FACS) to estimate microbial growth and phenotype (Zuleta et al., 2014; García-Timermans et al., 2020). However, characterizing cells within a culture without disturbing conditions (i.e., microorganisms live imaging; Bourges et al., 2020) while mimicking *in situ* pressures (from the deep biosphere) remains a challenge whenever high-pressure vessels are not equipped with a sapphire or optic window for cell characterization.

In this study, the high-pressure microfluidic screening approach yielded 40 growth curves at different pressure and temperature conditions in a record time.

**Figure 6:**
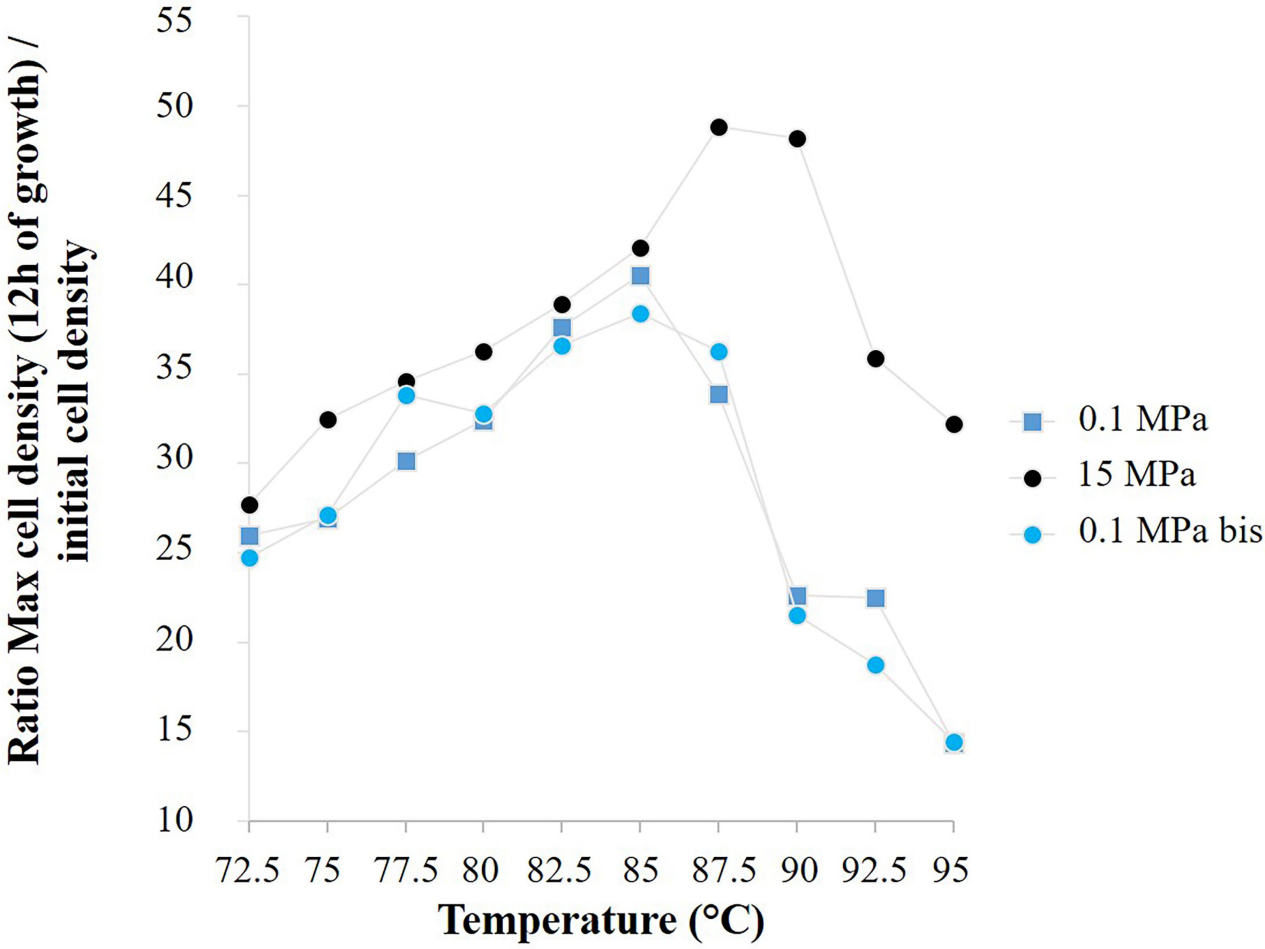
Ratio of the mean cell density of *Thermococcus barophilus* after 12 h of growth to the corresponding mean initial cell density, for three temperature gradient microfluidic experiments (72.5°C–95°C): atmospheric pressure (0.1 MPa) in a microchip of 1.5 nl pool volume, both 15 MPa and atmospheric pressure (0.1 MPa bis) in a microchip of 1.9 nl pool volume.

For *T. barophilus*, the final growth rates are comparable with the literature at ambient pressure and temperatures between 75°C and 90°C (Marteinsson et al., 1999) and at 85°C and for pressure conditions below 20 MPa (i.e., 0.35–0.45 h^−1^; Vannier et al., 2015). Marteinsson and colleagues reported that *T. barophilus* was an obligate piezophile for temperature above 95°C. Our study confirmed that *T. barophilus* shifts its tolerance to higher temperatures when growing under higher-pressure conditions (Figures 4, 6), and showed that it is still able to grow at atmospheric pressure and 95°C (well-defined environment with a precise temperature measurement, Figure 1; Supplementary Information 1). At atmospheric pressure, growth slowed as did cell density, which was the lowest at this extreme temperature. It is well-known in the literature that elevated pressures significantly increase the metabolism of deep-sea strains while growing at elevated temperatures (Miller et al., 1988; Takai et al., 2008). In addition, the high-pressure shifts the upper temperature range of hyperthermophilic strains (Miller et al., 1988; Pledger et al., 1994; Canganella et al., 1997; Marteinsson et al., 1999), which was also observed in this study (Figure 6). The culture approach is important for studying the upper temperature limit of life in hyperthermophilic microorganisms, and even more so when they are piezophilic, as high-pressure is an essential metabolic driving force for these strains isolated from the deep-sea (Takai et al., 2008; Oliver et al., 2020). In this study, combining several conditions of high temperature and pressure (without decompression) in a single experiment provided a rapid mean for characterizing the temperature range of a model strain of deep hydrothermal origin. Furthermore, this work demonstrates that high-pressure microfluidics is a robust and reproducible technique to examine microbial growth at different pressure conditions. Indeed, *T. barophilus* displayed the same temperature profile when growing in different micropools volume (1.5 and 1.9 nl; Figure 6).

Concerning *M. thermolithotrophicus*, this microfluidic setup allowed us to cultivate the methanogen strain under elevated pressure conditions (i.e., 5 and 10 MPa) with two different partial pressure conditions of H_2_/CO_2_ (i.e., 2 and 3 MPa, respectively). *M. thermolithotrophicus* cells were easily detected in the micropools using reflective mode (Visible), and can also be monitored thanks to their putative autofluorescence (Supplementary Figure S3). The growth of this strain is known to be enhanced under elevated pressure conditions (i.e., 50 MPa, see Bernhardt et al., 1988). Thus, we screened the effects of partial gas pressure (H_2_/CO_2_) on the cell yields, at two pressure conditions and over 10 temperature conditions. Interestingly, this strain seems to have a better growth under lower partial pressure of H_2_/CO_2_ over the range of temperature conditions tested (60°C–70°C). This study needs further investigations (e.g., gas solubility calculations according to the thermodynamic parameters) as well as to be confirmed with growth rate experiments in order to evaluate the toxicity of H_2_ and/or CO_2_ at higher partial pressures for this strain (Dupraz et al., 2013).

Beyond this first demonstration, this setup is adaptable and evolutive and will allow in the short term to cover a wider range of temperature and pressure conditions (0°C–150°C at up to 70 MPa with the new sapphire reactor technology; Marre et al., 2021) to better appreciate the growth (limits and tolerance) of a strain under extreme culture conditions. An interesting outcome is the implementation of various cultivation strategies in order to capture a wide variety of microorganisms with different metabolic requirements: (i) anaerobes and heterotrophs using a single-phase medium; (ii) autotrophs using a diphasic gas–liquid interface; and (iii) aerobes using a diphasic liquid–liquid interface where the immiscible oil serves as an oxygen supplier (Tanet et al., 2019; Figure 1). This microfluidic setup could provide new opportunities to screen known piezophiles and see how high pressure without decompression could extend the upper temperature range, e.g., for *T. barophilus* and other strict piezophiles such as *Pyrococcus yayanosii* CH1 (Zeng et al., 2009). In addition, high-pressure microfluidics could also be used as a fast-screening tool to characterize new isolates from the deep biosphere and to study the effect of pressure on growth in the vast majority of previously isolated strains in which this parameter has never been studied. Indeed, as an example, of the 129 bacterial species and 55 archaeal species that have been isolated from deep-sea hydrothermal vents to date and are recognized by the International Committee on Systematics of Prokaryotes (ICSP), only 23 have been examined for the effect of hydrostatic pressure on growth (Jebbar et al., 2015; Zeng et al., 2021).

Compared to conventionally studied batch microbial growth (i.e., large volume cultures at the ml or liter scale), microfluidics handles small volumes for batch culture (i.e., pl to μl) and such a volume variation could lead to a substantial change in microbial behavior when considering different situations such as surface area to volume ratio and nutritional aspect. The low cultivability scores in the laboratory of strains from natural environments have multiple explanations largely reviewed in the literature (e.g., Alain and Querellou, 2009; Lewis et al., 2021). Growth defects may be due to intrinsic environmental differences related, among others, to both accessible nutrients and the confined space available for growth. This variation in volume, known as the “bottle effect” is mainly reported in the literature for natural communities in aquatic environments (Hammes et al., 2010). Depending on the strain and its site of isolation, the effect of containment may be species-dependent and the size of the compartmentalization may trigger or constrain a microbial strain-specific phenotype. The model strain studied here, i.e. *T. barophilus*, comes from a deep hydrothermal vent, which is an environment with significant local chemical and physical gradients at the millimeter to the centimeter scale, changing in time and space, and containing a multitude of microniches for the growth of microorganisms (Dick, 2019). Microfluidics enables the handling of local gradients on a very small scale. It therefore appears to be a relevant cultivation approach to understand cell behavior (i.e., the microbial response to the local environment) or even cell interaction on a small scale. Such a confined environment for microbial cultivation does not account for larger scale processes (e.g., microbial dispersion and colonization) and depending on the type of study, multi-scale controls are needed to avoid biases induced by confined cultures. Here, *T. barophilus* and *M. thermolithotrophicus* cells were able to grow in a confined environment (mono- or diphasic) over several cell generations (about 2 log) in a similar fashion to dynamic growth in a “large” batch liquid culture (i.e., ml cultures in a closed vial). Thus, high-pressure microfluidics is a complementary approach to study microbial behavior under *in situ* pressure conditions.

Despite the reproducibility of this cultivation approach, such a confined environment presents a local nutrient gradient and motile microorganisms could use of their swimming abilities to seek out favorable available resources and/or conditions (Stocker and Seymour, 2012). *Thermococcus barophilus* is a motile microorganism but low-pressure conditions abolish its swimming capabilities (Vannier et al., 2015). Therefore, this approach on-a-chip is suitable at pressure conditions below 20 MPa, as *T. barophilus* cells are non-motile at these low pressure conditions and will not migrate from one pool to another. Concerning the application of this methodology to motile microorganisms, future developments will consider the use of non-miscible inert fluid (such as Fluorinert©) injected in the main microchannel to “seal” the micropools during the experiment, therefore preventing cell migration (see Figure 1 and the concerning protocol in the method section). Nevertheless, one can also think of taking advantage of connections between pore-like structures (e.g., porous media) on-a-chip with temperature and/or chemical gradients to get insights into cell motility towards better living conditions, which can be investigating in real time and under pressure with such approach. In summary, microspatial structures matter for phenotyping studies, thus it is important to design the appropriate microreactor in order to take into account variable parameters (e.g., cell migration, nutrient availability, diffusion coefficient) to ensure both the reproducibility and the closest representativeness of microbial habitats.

## Conclusion and Perspectives

This study highlighted the potentialities of high-pressure microfluidics for microbial strain phenotyping and validated high-pressure microfluidics as a valuable complementary tool for temperature range screening of extremophilic microorganisms, under pressure. It demonstrated that microfluidics can be used in order to accelerate microbial phenotyping of microorganisms (here piezo-hyper/thermophilic models) and provided a proof-of-concept for the operation, efficiency and reliability of microfluidics to screen several parameters at a time (e.g., temperature, pressure, and also nutrients gradient) and get the overall and combined phenotype of a single strain in a record time. This set up also demonstrated its versatility with the ability to target various metabolic requirements, reflecting the wide variety of the microbial metabolisms in the deep biosphere.

In this study, one pressure condition experiment (in quadruplicate) took only a few days (pre-culture plus cultivation test) to perform 10 temperature conditions whereas the conventional high-pressure microbiology approach would have taken weeks or months to get these data. High-pressure microfluidic technology can acquire large amounts of data in a short time, resulting in a huge amount of data to process (i.e., image capture, image analysis, and data processing). Our future goal is to implement automation of cell image capture and cell counting using artificial intelligence (machine learning in particular). Indeed, machine learning could help speed up counting but also increase accuracy and reduce errors (Qu et al., 2019). Such a methodology coupled to high-pressure microfluidics, would promote in-depth investigations (e.g., morphologies, catabolisms, and effects of chemicals) of various microorganisms from the deep-biosphere and greatly increase our knowledge of this remote biosphere.

Microfluidics provides an excellent opportunity to better document microbial phenotypes in a short time. This technology is widely applicable to other extremophiles (e.g., halophiles, psychrophiles) and could be adapted to diverse and more challenging culture conditions in order to explore the vast extremophilic microbial realm on Earth. The development of resilient microfabrication materials for extreme microfluidics (e.g., sapphire microreactors – (Marre et al., 2021)) is worth considering and could afford unique features to capture the large as-yet uncultured microbial fraction.

## Data Availability Statement

The original contributions presented in the study are included in the article/Supplementary Material, and further inquiries can be directed to the corresponding authors.

## Author Contributions

AC and SM designed this research project, made the microreactor design, analyzed data, and wrote the original draft. AC, SM, and ON developed the set-up. ON developed the temperature gradient device. AC and ML performed the experiments and collected data. AC, ON, and KA edited and wrote sections of the manuscript. All authors contributed to revisions of the manuscript, tables, and figures and approved the submitted version.

## Funding

Funding for this work was provided by the European Research Council (ERC) under the European Union’s Horizon 2020 research and innovation program (grant agreement no. 725100, project Big Mac).

## Conflict of Interest

The authors declare that the research was conducted in the absence of any commercial or financial relationships that could be construed as a potential conflict of interest.

## Publisher’s Note

All claims expressed in this article are solely those of the authors and do not necessarily represent those of their affiliated organizations, or those of the publisher, the editors and the reviewers. Any product that may be evaluated in this article, or claim that may be made by its manufacturer, is not guaranteed or endorsed by the publisher.
