# Supplementary material for: High-Pressure Microfluidics for Ultra-Fast Microbial Phenotyping
Source: Front Microbiol. 2022 May 23;13:866681. doi: 10.3389/fmicb.2022.866681 (PMC9168469; doi:10.3389/fmicb.2022.866681)
Supplement: Supplementary file 1 [file Data_Sheet_1.PDF]

Supplementary Material

Supplementary information 1: Temperature gradient system validation

The temperature gradient system was cautiously characterized through careful temperature measurements to ensure high accuracy of the temperature inside each micro-pool (**Fig. S1**).

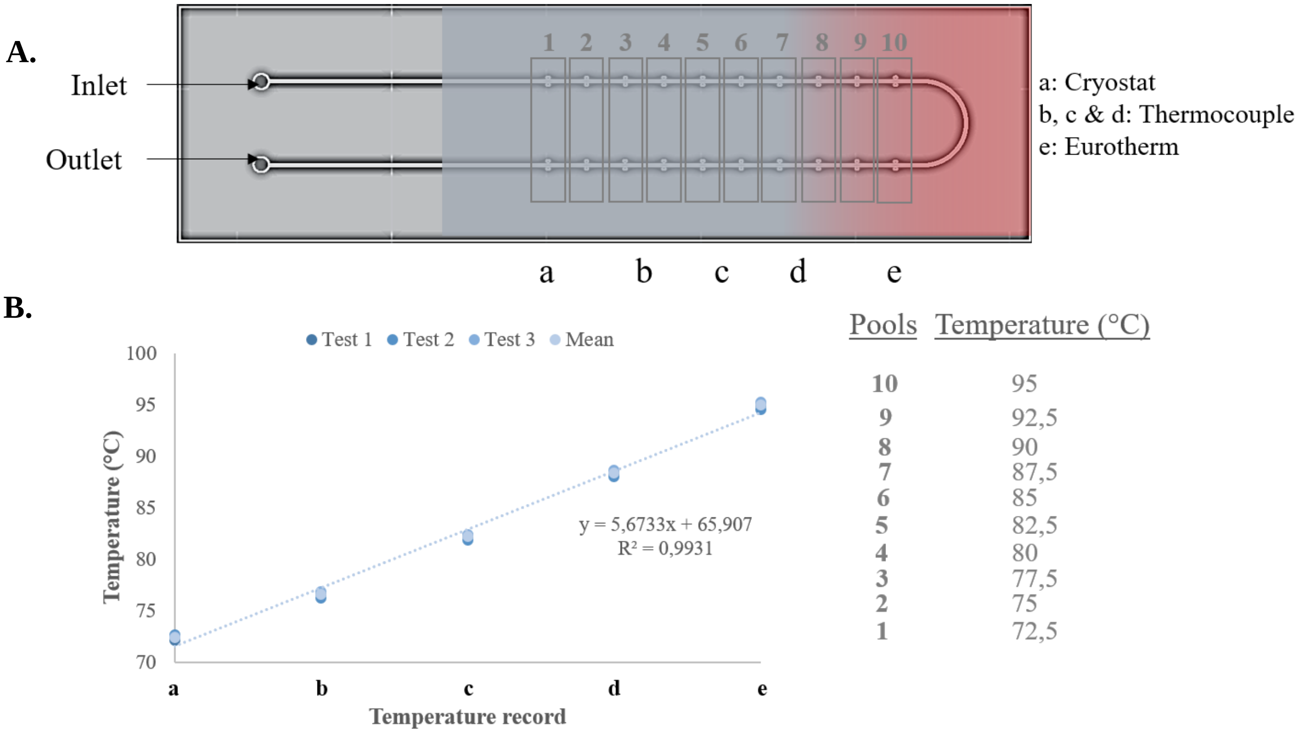

Figure S1: Example of the temperature gradient characterization in the case of *T. barophilus* phenotyping experiments. **(A)** Schematic of the temperature measurement on the microfluidic chip. The temperature was recorded three times with five data measurement: cryostat (a), three thermocouples at different places along the gradient (b, c and d) and a heating cartridge driven by an Eurotherm temperature controller (e). **(B)** Linear gradient along the microfluidic chip and temperatures corresponding to the quadruplicate pools.

Supplementary information 2: Growth curves of *T. barophilus*

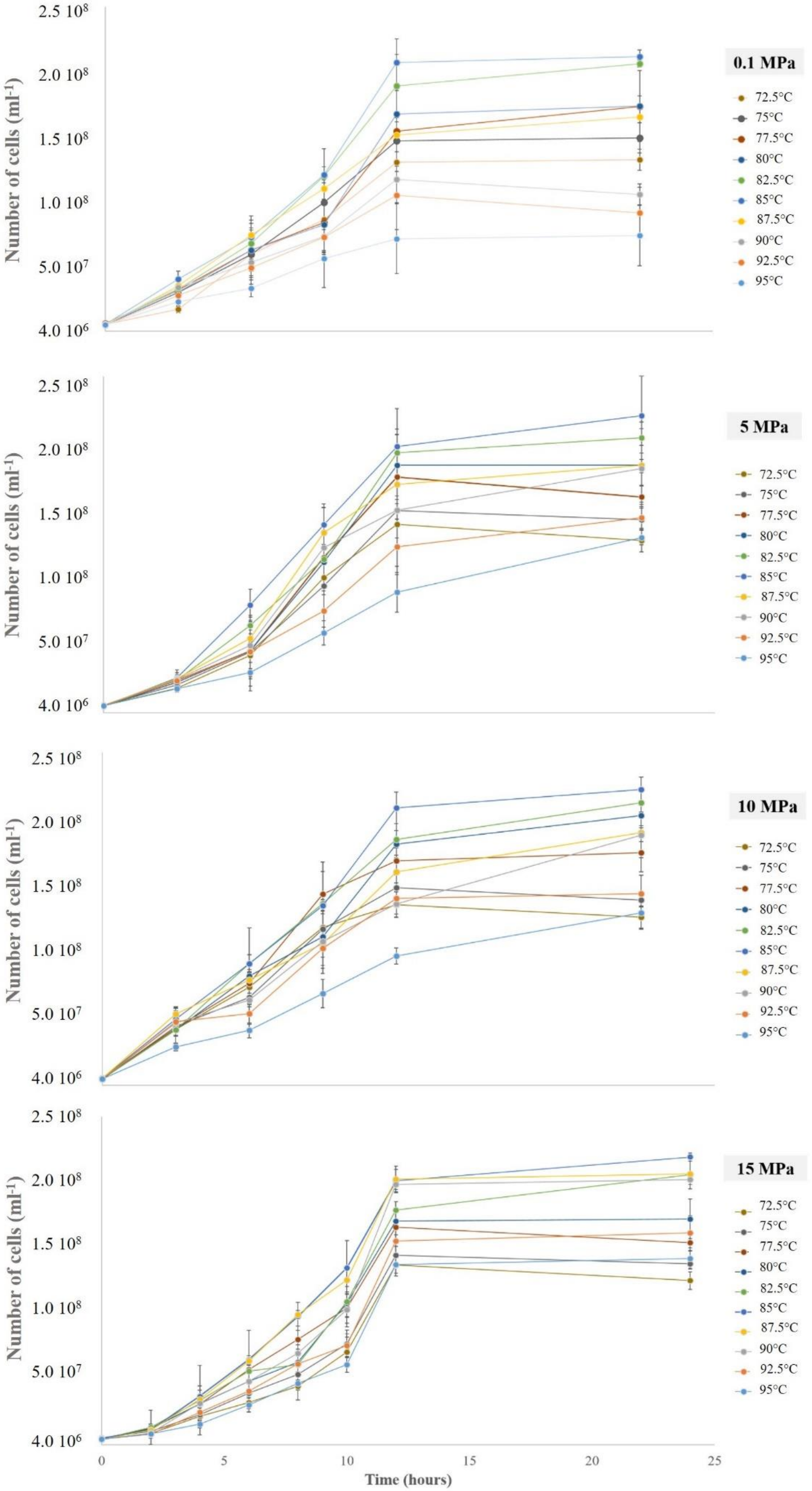

Figure S2: Growth curves of *T. barophilus* according to 10 different temperatures (72.5 to 95°C) at four pressure conditions (0.1, 5, 10 & 15 MPa). Data measurement are means and standard errors of quadruplicate growth experiments in the microfluidic pools.

Supplementary information 3: Methodology for image analysis

The cell concentration (cells.mL^{-1}) was calculated by: (1) determining precisely each micro-pool volume and (ii) assessing the number of cell in each micro-pool using semi-automatic counting with ImageJ and OpenCFU softwares, as detailed below:

(i) Micro-pool volume determination

Using ImageJ software, each pool picture (40 micropools on each microfluidic chip) was first converted to grayscale, set to the accurate scale and then processed to a binary image. The threshold was adjusted to measure only highlighted pixels within the area of interest. Binary images were eroded to have a circular frame (*i.e*. no channel measurement) thus the overall top and bottom area could be determined. The area was then correlated to the measured depth of the pools along with the slope of the micro-pool boundaries (which is inherent to the wet etching process used to fabricate the microreactor) to obtain precisely the pool volume values.

(ii) Cell counting methodology

For each obtained image, the OpenCFU software was used. A regular threshold between 2 and 5 (depending on the image contrast light) was applied to decrease noise and background variations. The resolution of the obtained images is 5.5 pixels / µm. Given that the size of both *T. barophilus* and *M. thermolithotrophicus* cells can typically range from 0.8 to 2 µm, a minimum radius (size in pixel) of 2 and a maximum one of 6 was applied for the automatic detection in order to exclude big sulfur particles in the case of TRM growth media. Meanwhile, a coarseness of 0.1 was chosen to recognize both species (lighter than particles). Errors were then corrected manually after careful analysis.

Supplementary information 4: *Methanothermococcus thermolithotrophicus* experiments

A.

B.

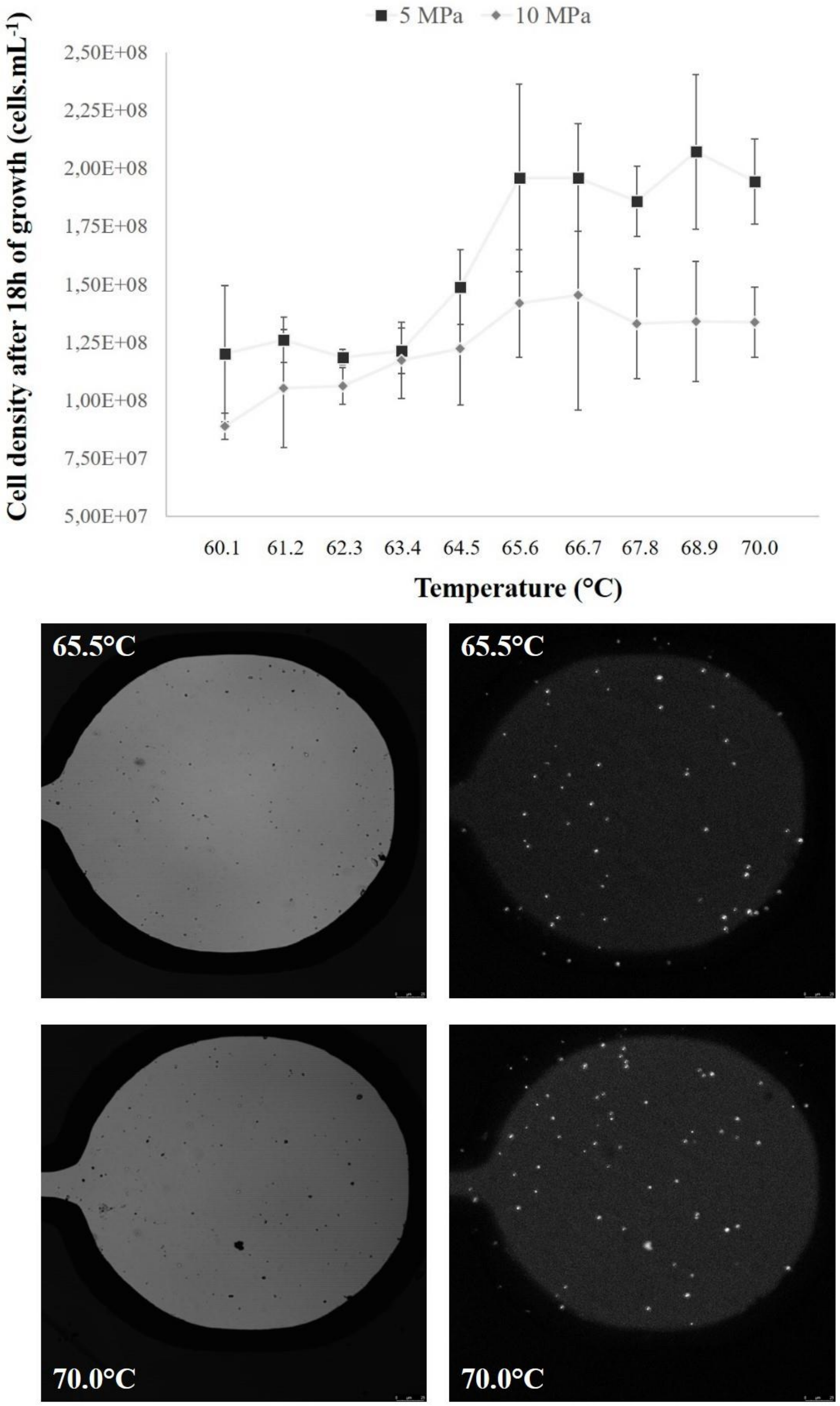

Figure S3: (A.) *M. thermolithotrophicus* cell yields after 18h of growth over 10 temperature conditions (*i.e.* 60.1 to 70°C) for two pressure conditions, while growing in the temperature gradient on-a-chip. Error bars represent the standard deviation from the four replicates on a single experiment using the gradient on chip microfluidic setup. (B) Pictures of *M. thermolithotrophicus* cells growing at 5 MPa, 65.5 and 70°C, after 8h of growth (isolated experiment). Left panel are reflection mode (Visible) pictures and right panel are fluorescence pictures (λ_{ex}=405 nm – λ_{em}=420nm) taken with the confocal microscope. Bar scales on the bottom right of the pictures indicates 25 µm.
